# Navigating surface reconstruction of spinel oxides for electrochemical water oxidation

**DOI:** 10.1038/s41467-023-38017-3

**Published:** 2023-04-28

**Authors:** Yuanmiao Sun, Jiarui Wang, Shibo Xi, Jingjing Shen, Songzhu Luo, Jingjie Ge, Shengnan Sun, Yubo Chen, John V. Hanna, Shuzhou Li, Xin Wang, Zhichuan J. Xu

**Affiliations:** 1 Faculty of Materials Science and Energy Engineering/Institute of Technology for Carbon Neutrality, Shenzhen Institute of Advanced Technology, Chinese Academy of Sciences, Shenzhen, 518055 P. R. China; 2 Shenzhen Key Laboratory of Energy Materials for Carbon Neutrality, Shenzhen Institute of Advanced Technology, Chinese Academy of Sciences, Shenzhen, 518055 P. R. China; 3 School of Materials Science and Engineering, Nanyang Technological University, 50 Nanyang Avenue, Singapore, 639798 Singapore; 4 Singapore-HUJ Alliance for Research and Enterprise (SHARE), NEW-CREATE Phase II, Campus for Research Excellence and Technological Enterprise (CREATE), Singapore, 138602 Singapore; 5 Institute of Chemical and Engineering Science, Agency for Science Technology and Research (A*Star), Singapore, 627833 Singapore; 6 Department of Chemical and Biological Engineering, HKUST Jockey Club Institute for Advanced Study, Energy Institute, The Hong Kong University of Science and Technology, Clear Water Bay, Kowloon Hong Kong, China; 7 Institute of Materials Research and Engineering (IMRE), Agency for Science, Technology and Research (A*Star), 2 Fusionopolis Way, Innovis #08-03, Singapore, 138634 Republic of Singapore; 8 Department of Physics, University of Warwick, Coventry, UK; 9 Department of Chemistry, City University of Hong Kong, Hong Kong, China; 10 Energy Research Institute @ Nanyang Technological University, ERI@N, Interdisciplinary Graduate School, Nanyang Technological University, 50 Nanyang Avenue, Singapore, 639798 Singapore; 11 Center for Advanced Catalysis Science and Technology, Nanyang Technological University, 50 Nanyang Avenue, Singapore, 639798 Singapore

**Keywords:** Electrocatalysis, Catalysis, Electrocatalysis

## Abstract

Understanding and mastering the structural evolution of water oxidation electrocatalysts lays the foundation to finetune their catalytic activity. Herein, we demonstrate that surface reconstruction of spinel oxides originates from the metal-oxygen covalency polarity in the M_T_–O–M_O_ backbone. A stronger M_O_–O covalency relative to M_T_–O covalency is found beneficial for a more thorough reconstruction towards oxyhydroxides. The structure-reconstruction relationship allows precise prediction of the reconstruction ability of spinel pre-catalysts, based on which the reconstruction degree towards the in situ generated oxyhydroxides can be controlled. The investigations of oxyhydroxides generated from spinel pre-catalysts with the same reconstruction ability provide guidelines to navigate the cation selection in spinel pre-catalysts design. This work reveals the fundamentals for manipulating the surface reconstruction of spinel pre-catalysts for water oxidation.

## Abstract (web summary)

Precise control of water oxidation activity calls for the mastery of surface reconstruction of electrocatalysts. Here, the authors identify the structural origin of surface reconstruction on spinel oxides and report a criterion to evaluate the reconstruction degree, which enable the design of spinel pre-catalyst with controlled reconstruction ability.

## Introduction

The increasing global energy consumption and depletion of fossil fuels have necessitated the establishment of a sustainable and environmentally friendly energy cycle. Hydrogen has been expected to be an ideal solution because of its renewability, high energy density, and clean exhaust products^1^. Among various hydrogen production techniques, electrochemical water splitting offers a green method to directly generate hydrogen using electricity, which can be acquired from renewable sources such as wind, tides, and sunlight. However, the overall efficiency of water splitting is largely hindered by the sluggish oxygen evolution reaction (OER) at the anode, which makes the design of efficient OER catalysts an urgent issue^2,3^.

Rational catalyst design calls for a comprehensive understanding of the reaction process, based on which key reaction fundamentals can be summarized to aid the design of remarkable catalyst. For example, it has been long recognized that the adsorption of reaction intermediates on the active sites plays important role in determining the activity^3–5^. Based on this, oxide IrO_x_/SrIrO_3_ and perovskite Ba_0.5_Sr_0.5_Co_0.8_Fe_0.2_O_3–d_ (BSCF) have been designed, which exhibit neither too strong nor too weak binding affinity to the oxygen-related intermediates^6,7^. More recently, the surface reconstruction of electrocatalysts under electro-oxidation conditions has been widely detected^8–10^. Surface reconstruction leads to in situ formation of a core-shell structure, in which the oxyhydroxides in the surface and near-surface region are considered as the real catalytic species for the reaction. This paves the way for new design paradigm to manipulate the surface reconstruction of pre-catalyst for advanced OER activity. However, to date studies about surface reconstruction mainly focus on exploring the compositional effect on the activity of the reconstructed oxyhydroxides;^11,12^ it remains unclear in which way and to what extent we can control the surface reconstruction of pre-catalyst.

A prerequisite to manipulate surface reconstruction is to identify what triggers the process of surface reconstruction. Some pioneer works recommend cation leaching as the origin of surface reconstruction^8,13,14^. For example, lithium cation is considered unstable in alkaline conditions and therefore can easily leach out to activate surface reconstruction. J. Wang et al. have achieved severe in situ reconstruction in layered LiCoO_2–x_Cl_x_ (x = 0, 0.1 and 0.2) due to lithium leaching^14^. However, no such reconstruction was observed in spinel LiMn_2_O_4_, where lithium cations are intensively involved but the spinel framework remains almost unchanged during OER^15,16^. The contradictory phenomena suggest that the leaching of metal cations is not element-dependent but should be material-dependent, which necessitates deeper investigations into the electronic structures to give an explicit explanation. Unfortunately, this part has been less touched and remains elusive. Besides, precise control of the reconstruction ability of pre-catalyst is demanded. Surface reconstruction may not always bring activity enhancement. If the intrinsic activity of pre-catalyst is superior to that of the reconstructed counterpart, strategies should be made to suppress the reconstruction to guarantee a long reaction durability. On the contrary, if the reconstructed oxyhydroxide shows superior activity, the pre-catalyst should be designed with high reconstruction ability to promote surface reconstruction. This calls for the establishment of a structure-reconstruction relationship, based on which the reconstruction ability of pre-catalyst can be forecasted and finetuned. In addition, principles are needed to guide the selection of metal cations in the design of pre-catalyst. The activity of the reconstructed oxyhydroxides is dependent on the type and ratio of metal cations^11^. Therefore, to facilitate in situ generation of highly active oxyhydroxides, investigations are intensively needed to pinpoint the active cation species in reconstructed oxyhydroxides.

Herein, using spinel Li_x_Co_1–x_Co_2_O_4_ as model catalysts, we demonstrate that surface reconstruction of spinel oxides originates from the biased metal-oxygen covalency between MO_4_ and MO_6_. Combining computational and experimental approaches, a structure-reconstruction relationship has been established, based on which the reconstruction ability of spinel pre-catalyst can be effectively forecasted. Further electrochemical analysis and computational calculations deliver some useful clues for the cation selection in spinel pre-catalysts. This work unveils the structural origin of reconstruction for spinel oxides. It also provides a structure-reconstruction relationship applicable to all spinel family to forecast their reconstruction ability. The demonstrated strategies to master surface reconstruction provides firm theoretical foundations to navigate the design of spinel pre-catalysts with controllable reconstruction ability for electrochemical water oxidation.

## Results and discussions

### Theoretical investigation of the structural origin of surface reconstruction on spinel oxides

**Fig. 1:**
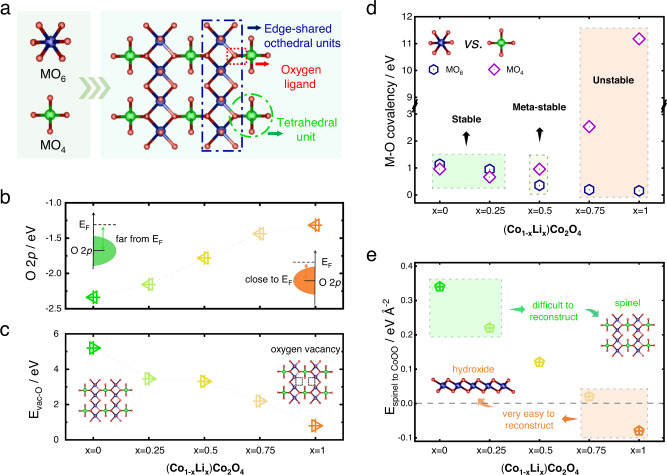
DFT investigations of the electronic structure evolution of spinel (Co_1−x_Li_x_)Co_2_O_4_ (x = 0, 0.25, 0.5, 0.75 and 1). **a** Illustration of the spinel AB_2_O_4_ structure that composed of tetrahedral units (MO_4_) and octahedral units (MO_6_). **b** Calculated oxygen p-band center of spinel (Co_1−x_Li_x_)Co_2_O_4_ (x = 0, 0.25, 0.5, 0.75 and 1). **c** Calculated vacancy formation energy (E_O-vac_) of spinel (Co_1−x_Li_x_)Co_2_O_4_ (x = 0, 0.25, 0.5, 0.75 and 1). **d** Metal-oxygen covalency of the MO_4_ and MO_6_ units in spinel (Co_1−x_Li_x_)Co_2_O_4_ (x = 0, 0.25, 0.5, 0.75 and 1). **e** The surface transformation energy (E_spinel to CoOO_) from spinel to layered species.

The structural composition of spinel oxides is AB_2_O_4_, in which the cations in A sites and B sites are tetrahedrally and octahedrally coordinated, respectively. Within the structure, the octahedral units connect to one another via an edge-shared form, while the connection between tetrahedral units and octahedral units is corner-shared (Fig. 1a). The oxygen ligands are arranged in a cubic close-packed lattice and each of them is shared by three octahedral cations and one tetrahedral cation to create a M_T_-O-M_O_ backbone^17^. When catalyzing OER, the spinel frame may collapse and reconstruct to amorphous oxyhydroxides during electrochemical cycling. The evolution from spinel to amorphous oxyhydroxides necessitates the breakage between the tetrahedral and octahedral units, during which highly reactive and transferrable oxygen ligands are believed to facilitate the phase transformation. Besides, because the cations in the reconstructed oxyhydroxides stay in an octahedral coordination environment, a stronger metal-oxygen covalency of the octahedral units than the tetrahedral units may be advantageous for the reconstruction process. In other words, a biased M_T_-O-M_O_ backbone with oxygen binds more strongly to the octahedral cations may be decisive for more smoothly and thoroughly reconstructed oxyhydroxides.

To verify above hypotheses, a case study is performed on spinel (Co_1−x_Li_x_)Co_2_O_4_ (x = 0, 0.25, 0.5, 0.75 and 1). Spinel (Co_1−x_Li_x_)Co_2_O_4_ is chosen because the CoO_6_ units are the only catalytically active centers after reconstruction, which allows us to explicitly analyze the degree of surface reconstruction. The density functional theory (DFT) calculations are carried out to explore the electronic structure evolution with the change of spinel composition. As a prerequisite for structural collapse, the reactivity of oxygen ligands is first examined. With the increase of lithium in spinel (Co_1−x_Li_x_)Co_2_O_4_, the oxygen *p*-band center gradually increases and moves closer to the Fermi level (Fig. 1b), indicating the oxygen ligands are becoming more active. Meanwhile, the calculated oxygen vacancy formation energy (E_vac-O_) of CoCo_2_O_4_ is as high as 5.20 eV, suggesting severe difficulty of generating an oxygen vacancy in the structure. With the tetrahedral cobalt cations being replaced by lithium cations, the energy required to generate oxygen vacancy steadily decreases (Fig. 1c). The value of E_vac-O_ decreases to 0.80 eV in LiCo_2_O_4_, implying high mobility of oxygen ligands to escape from the spinel lattice. The trends of oxygen *p*-band center and oxygen vacancy formation energy demonstrate an activation effect to the oxygen ligands with the increase of lithium in spinel (Co_1−x_Li_x_)Co_2_O_4_.

The M–O bond strength of the M_T_-O-M_O_ backbone is subsequently evaluated by calculating the metal-oxygen covalency in the spinel structure. The metal-oxygen covalency is defined as the difference in band center between oxygen *p*-orbitals and metal *d*-orbitals, in which a higher value represents a weaker covalency^17^. As shown in Fig. 1d, the metal-oxygen covalency of tetrahedral units and octahedral units are quite close to each other in spinel CoCo_2_O_4_ and (Co_0.75_Li_0.25_)Co_2_O_4_. In this case, oxygen binds equally to the tetrahedral and octahedral cations, which fabricates a nonpolar M_T_-O-M_O_ backbone. The spinel frame is therefore stable and should be difficult to collapse for reconstruction. However, with more lithium cations being introduced into the spinel lattice, the metal-oxygen covalency of tetrahedral and octahedral units begins to get weakened and strengthened, respectively. In spinel (Co_0.5_Li_0.5_)Co_2_O_4_, the covalency of MO_4_ units is weaker than that of MO_6_ units, suggesting a biased M_T_-O-M_O_ has evolved. Compared to CoCo_2_O_4_ and (Co_0.75_Li_0.25_)Co_2_O_4_, this spinel structure becomes meta-stable and the process of oxyhydroxides generation may be facilitated. The metal-oxygen covalency gap between MO_4_ and MO_6_ is further widened in spinel (Co_0.25_Li_0.75_)Co_2_O_4_ and LiCo_2_O_4_. In spinel LiCo_2_O_4_, the calculated covalency gap is as high as 11.03 eV, implying the spinel frame is highly unstable and the reconstruction to octahedrally coordinated oxyhydroxides is considerably feasible. To further investigate the trend of surface reconstruction ability in spinel (Co_1−x_Li_x_)Co_2_O_4_, the surface transformation energy from spinel to layered oxide is calculated. The surface transformation energy is defined as the energy of (111) spinel surface relative to that of the layered CoOO species (more details are summarized in Methods). As shown in Fig. 1e, the surface transformation energy of spinel CoCo_2_O_4_ and (Co_0.75_Li_0.25_)Co_2_O_4_ is far above 0 eV, suggesting the transformation from spinel to layered species is difficult. With more lithium cations substituting cobalt cations, the energy (per surface area) needed to transform spinel to layered structure gradually decreases. This indicates the difficulty for surface reconstruction is reduced. In spinel (Co_0.25_Li_0.75_)Co_2_O_4_ and LiCo_2_O_4_, the surface transformation energy is quite close to and even smaller than 0 eV, hinting very easy-going surface reconstruction. Therefore, we can rationally deduce that the increase of lithium cations in spinel (Co_1−x_Li_x_)Co_2_O_4_ will result in enhanced surface reconstruction ability.

### Synthesis and structural characterizations of spinel Li_x_Co_3−x_O_4_

**Fig. 2:**
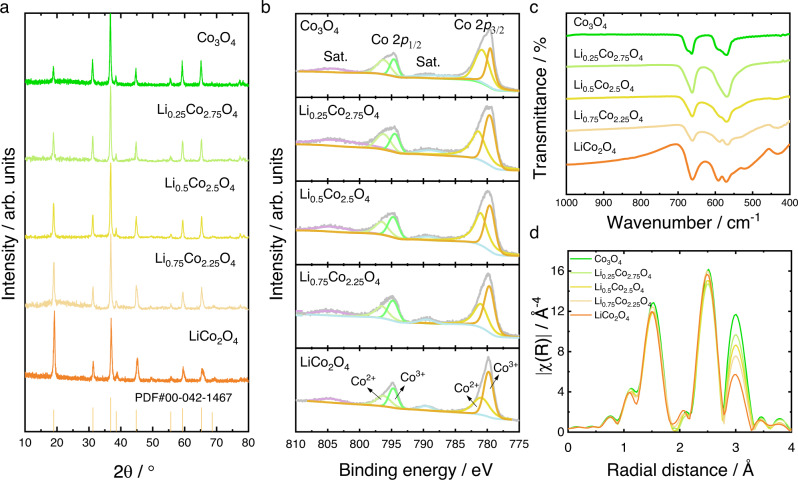
Structural characterization of the as-prepared spinel Li_x_Co_3−x_O_4_. **a** X-ray diffraction (XRD) patterns of the as-prepared spinel Li_x_Co_3−x_O_4_ samples. **b** The Co 2*p* X-ray photoelectron spectroscopy (XPS) spectra of the as-prepared spinel Li_x_Co_3−x_O_4_ samples. **c** The Fourier Transform Infrared (FTIR) spectra of the as-prepared spinel Li_x_Co_3−x_O_4_ samples. **d** Fourier-transformed k3-weighted Co K-edge X-ray absorption fine structure (EXAFS) spectra of the as-prepared spinel Li_x_Co_3−x_O_4_ samples.

To verify the reconstruction trend predicted by DFT calculations, spinel Li_x_Co_3−x_O_4_ (x = 0, 0.25, 0.5, 0.75, and 1) were prepared by a nitrate decomposition method followed by heat treatment (more details are summarized in Methods). The inductively coupled plasma optical emission spectrometry (ICP-OES) results (Supplementary Table 1) confirm that all the as-prepared samples remain stoichiometric Li_x_Co_3−x_O_4_. The Li 1 *s* XPS spectra of the pristine samples and the derived lithium molar ratio are summarized in Supplementary Figure 1. The results evidence that, although slightly leached, the surface lithium concentration still obeys the same trend as the total lithium concentration. The crystal structure of the as-prepared spinel Li_x_Co_3−x_O_4_ was confirmed by X-ray diffraction (XRD) analysis. As shown in Fig. 2a, the diffraction peaks of the as-prepared samples match well with that of the standard Co_3_O_4_ (PDF#00-042-1467), indicative of typical cubic spinel structure. The peak at 19° gets intensified with the increase of lithium in spinel Li_x_Co_3−x_O_4_, suggesting the gradual decrease of tetrahedral cobalt cations and that the introduced lithium cations mainly reside in the tetrahedral sites^18^. The X-ray photoelectron spectroscopy (XPS) was performed to further evaluate the surface Co^2+^/Co^3+^ ratio of the samples. The Co 2*p* spectrum exhibits two major peaks at 795 eV and 780 eV, ascribed to the typical Co 2*p*_1/2_ and Co 2*p*_3/2_ orbitals, respectively. An approximate energy variation of 15 eV is observed between the two major peaks, consistent with that reported in previous studies^19,20^. The deconvolution of the two major peaks shows two characteristic peaks, which can be ascribed to Co^2+^ and Co^3+^, respectively. As displayed in Fig. 2b, with more lithium cations substituting cobalt cations, the relative intensity of Co^2+^ characteristic peak gradually decreases, indicating the decrease of Co^2+^ cations and an increase of cobalt oxidation state. The quantitative atomic ratio of Co^2+^/Co^3+^ could be estimated by comparing the area of the characteristic peaks^19^. The results, which are given in Supplementary Table 2, show an obvious decrease of the surface Co^2+^/Co^3+^ ratio, further hinting the occupation of lithium cations in the tetrahedral sites.

The Fourier Transform Infrared (FTIR) spectroscopy was employed to probe the local chemical bond change of the as-prepared samples (Fig. 2c). The bands in the frequency range from 500 cm^−1^ to 700 cm^−1^ are attributed to the vibrations of the CoO_6_ octahedra^21^. Specifically, the one at around 570 cm^−1^ is attributed to the asymmetric stretching modes of CoO_6_ and the one at around 670 cm^−1^ to the bending modes of Co-O-Co^21^. The band at around 448 cm^−1^ corresponds to the stretching vibrations of LiO_4_ tetrahedra^22^. This characteristic band gets intensified with the increase of Li in spinel Li_x_Co_3−x_O_4_, which indicates, once again, the residence of lithium cations in the tetrahedral sites. The cation occupancy was also confirmed by the extended X-ray absorption fine structure (EXAFS) technique. Figure 2d displays the Fourier-transformed *k*^3^-weighted Co K-edge EXAFS spectra of the as-prepared samples. The peaks at 1.5 Å and 2.5 Å correspond to Co-O scattering and Co_Oct_-Co_Oct_ scattering, respectively. The peak at 3.0 Å corresponds to the Co_Tet_-Co_Oct_ scattering. With more lithium substituting cobalt, the intensity of Co_Tet_-Co_Oct_ scattering peak steadily decreases, suggesting the gradual absence of tetrahedral cobalt in spinel Li_x_Co_3−x_O_4_ and therefore implying the occupation of lithium cations in tetrahedral sites. It can be rationally concluded that the as-prepared spinel Li_x_Co_3−x_O_4_.

### Electrochemical analysis of the pristine and fully cycled spinel Li_x_Co_3−x_O_4_

**Fig. 3:**
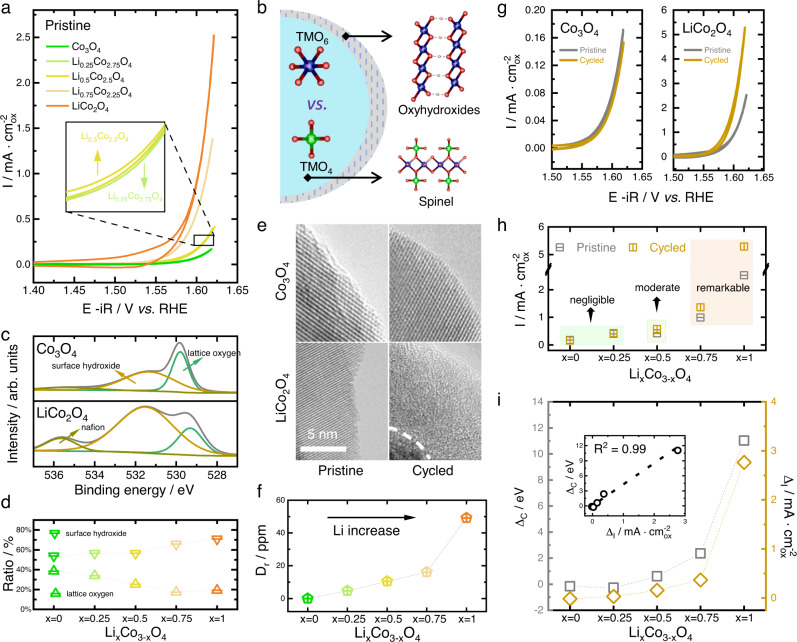
Electrochemical analysis of the pristine and fully cycled spinel Li_x_Co_3−x_O_4_. **a** The cyclic voltammetry (CV) curves of the pristine Li_x_Co_3−x_O_4_. **b** Schematic of the surface reconstruction of spinel oxides. **c** The O 1 *s* spectra of fully cycled spinel Co_3_O_4_ and LiCo_2_O_4_. **d** The relative ratio of surface oxyhydroxide and lattice oxygen in the fully cycled spinel Li_x_Co_3−x_O_4_. **e** The high-resolution transmission electron microscopy (HRTEM) images of the pristine and cycled spinel Co_3_O_4_ and LiCo_2_O_4_. **f** The estimated reconstruction degree (D_r_) of spinel Li_x_Co_3−x_O_4_. **g** The CV curve of the pristine and cycled spinel Co_3_O_4_ and LiCo_2_O_4_. **h** The experimentally measured current density (@ 1.62 V *vs*. RHE) of the pristine and cycled spinel Li_x_Co_3−x_O_4_. **i** The plot integrating both the experimentally measured and theoretically calculated reconstruction degree. The current density (@ 1.62 V *vs*. RHE) change (Δ_I_) between cycled and pristine spinel Li_x_Co_3−x_O_4_ is used to represent the experimentally measured reconstruction degree, while the DFT calculated metal-oxygen covalency polarity (the metal-oxygen covalency difference between MO_4_ and MO_6_, Δ_C_) is used to represent the theoretically predicted reconstruction degree. The insert shows the linear fitting of the two parameters.

After confirming the crystal structure and cation occupancy of the as-prepared samples, the electrochemical analysis was conducted. The electrochemical measurements were carried out within a potential window of ~1.0–1.62 V (*vs*. RHE) in 1 M KOH, with the catalytic activities being normalized by the surface area of oxides (more details are summarized in Methods). The surface area of oxides was obtained by Brunauer-Emmett-Teller (BET) technique, which has been proved to be suitable for measuring the surface area of metal oxides in powder form^23^. The current efficiency of spinel LiCo_2_O_4_ is 95.4 ± 2.5% (Supplementary Figure 3), confirming that the measured current mainly originates from OER. Figure 3a summarizes the iR-compensated activity of the pristine samples. The non-iR compensated results are shown in Supplementary Figure 4. The activity rises with more lithium cations substituting cobalt cations, probably because of the strengthened Co_Oct_-O covalency^24^. As the electrochemical cycling continues, the surface and near-surface region of the spinel structure may collapse and evolve into amorphous oxyhydroxides (Fig. 3b). The structural information and OER activity of the fully cycled spinel Li_x_Co_3−x_O_4_ were analyzed after performing the electrochemical cycling for 500 times. The XRD and FTIR results of the cycled samples are shown in Supplementary Figure 5, which evidences that the bulk of the samples remains spinel crystal and implies that the reconstruction process mainly occurs on the surface region. To probe the elemental composition of the near surface region after reconstruction, the Co 2*p* and Li 1 *s* XPS spectra of the cycled samples are recorded (Supplementary Figure 6). As illustrated, no Li 1 *s* spectra can be recorded, suggesting the absence of lithium in the near surface region. Besides, of all the samples, the positions of Co 2*p* characteristic peaks are quite close to those of CoOOH, which further evidences the formation of surface cobalt oxyhydroxide. Therefore, we believe the active sites after reconstruction should be the CoO_6_ units. The O 1 *s* XPS spectra were recorded to investigate the components of various oxygen species in the surface region. As exhibited in Fig. 3c, the relative ratio of lattice oxygen and surface oxyhydroxide becomes much smaller and higher, respectively, with cobalt being gradually substituted by lithium. The numerical ratios can be extracted by calculating the area the characteristic curve covers, which are given in Fig. 3d. The results evidently demonstrate the steady growth of surface oxyhydroxide with more lithium being present inside the spinel lattice, suggesting higher surface reconstruction degree with the increase of lithium cations. The comparison of O 1 *s* XPS spectra between the pristine and cycled samples are shown in Supplementary Figure 7. As demonstrated, compared to the pristine samples, all the cycled ones possess increased ratio of surface oxyhydroxide and decreased ratio of lattice oxygen, suggesting the destruction of spinel structure and the in situ formation of oxyhydroxides. Besides, more lithium incorporation is found to promote the generation of more surface oxyhydroxide, which further evidences the intensified surface reconstruction in high lithium-containing Li_x_Co_3-x_O_4_. The surface reconstruction can be visually characterized by high-resolution transmission electron microscopy (HRTEM) images. As given in Fig. 3e, the pristine and cycled HRTEM images of Co_3_O_4_ display smooth and crystalized surface termination, indicating the absence of surface reconstruction. By contrast, remarkable surface amorphization is observed in the cycled HRTEM image of LiCo_2_O_4_, reflecting considerable reconstruction at the surface region. The surface reconstruction degree (D_r_) can be evaluated by analyzing the volumetric ratio between the reconstructed portion and the totally loaded one, which can be obtained using the following equation (detailed derivation procedures of the equation is summarized in Methods):

1 $${D}_{r}=\frac{{V}_{{reconstruction}}}{{V}_{{loading}}}=\frac{\frac{{c}_{{Li}}{{\cdot }}{N}_{A}}{6.94{{\cdot }}x}{{\cdot }}{V}_{{mol}}}{\frac{4}{3}{{\cdot }}\pi {{\cdot }}{\left(\sqrt{\frac{{m}_{{loading}}{{\cdot }}{BET}}{4\pi }}\right)}^{3}}.$$

where ${c}_{{Li}}$, ${N}_{A}$, ${V}_{{mol}}$, $x$, ${m}_{{loading}}$, and ${BET}$ represent the content of Li in the cycled electrolyte, the Avogadro constant, the molecular volume of spinel Li_x_Co_3−x_O_4_, the stochiometric number x in spinel Li_x_Co_3−x_O_4_, the mass loading, and the BET area of the oxide, respectively. As displayed in Fig. 3f, a noticeable increase of reconstruction degree is observed with the increase of lithium cations in spinel Li_x_Co_3−x_O_4_. The trend further evidences the intensification of surface reconstruction in high lithium-containing Li_x_Co_3−x_O_4_.

The change in OER activity gives a more precise evaluation of the surface reconstruction degree. As above-mentioned, all the prepared samples share the same catalytically active centers after reconstruction, i.e., the reconstructed CoO_6_ units. This lays the foundation that the activity change between reconstructed catalyst and the pristine one can be convictive for assessing the surface reconstruction degree. The cyclic voltammetry (CV) curves of all the pristine and cycled samples are shown in Supplementary Fig. 8. It is obvious that the introduction of lithium is beneficial for a more apparent catalytic enhancement, as Co_3_O_4_ exhibits almost unchanged CV curves while LiCo_2_O_4_ shows dramatic rise after fully cycling (Fig. 3g). It’s worth to note that, based on the chronoamperometry results of spinel Co_3_O_4_ and LiCo_2_O_4_ (Supplementary Fig. 9), the incorporation of lithium in spinel structure does not influence the OER durability, as both catalysts exhibit rather stable catalytic performance within 60 operating hours. Figure 3h provides a direct comparison of the current density (@ 1.62 V *vs*. RHE) between the pristine and the cycled one. Similar to that illustrated in Fig. 1d, three distinct regions can be identified. For spinel Co_3_O_4_ and Li_0.25_Co_2.75_O_4_, the highly overlapped current density suggests negligible surface reconstruction. With more lithium being introduced into spinel Li_x_Co_3−x_O_4_, the change in current density becomes noticeable. In Li_0.5_Co_2.5_O_4_, the current density of cycled catalyst surpasses that of the spinel pre-catalyst, indicating the surface reconstruction is moderate. In spinel Li_0.75_Co_2.25_O_4_ and LiCo_2_O_4_, notable activity improvement can be seen, which signifies remarkable surface reconstruction. It’s worth to note that the surface area of samples is expected to increase after reconstruction, which can be evidenced by the measured electrochemically active surface area (ECSA) of the pristine and cycled samples (Supplementary Fig. 10). As exhibited in Supplementary Fig. 10a, the increase of ECSA gets intensified with the increase of lithium in pre-catalyst. This reveals the increased active sites, and therefore demonstrates the higher amorphization degree, in high lithium-containing samples. However, the increase of surface area does not alter the activity trend of the cycled samples (Supplementary Fig. 10b). To directly visualize the reconstruction degree in CV and compare the activities of the pristine and cycled samples within the same context, the current densities of the cycled samples are still normalized by the surface area of the pre-catalysts. By plotting the experimental activity enhancement (the change of current density @ 1.62 V *vs*. RHE, Δ_I_) and the calculated metal-oxygen covalency polarity of spinel (the metal-oxygen covalency difference between MO_4_ and MO_6_, Δ_C_) in the same pattern, a reasonably similar trend is present (Fig. 3i). The fitting between these two parameters shows a R square value of 0.99 (Fig. 3i insert), which strongly demonstrates that the metal-oxygen covalency polarity is reasonable to explain, and therefore can be used to predict, the surface reconstruction ability of spinel oxides. In other words, a structure-reconstruction relationship of spinel oxides can be outlined, i.e., higher metal-oxygen covalency polarity between MO_4_ and MO_6_ can result in stronger surface reconstruction, and vice versa.

### Guidelines for the design of highly active oxyhydroxides from spinel pre-catalysts

**Fig. 4:**
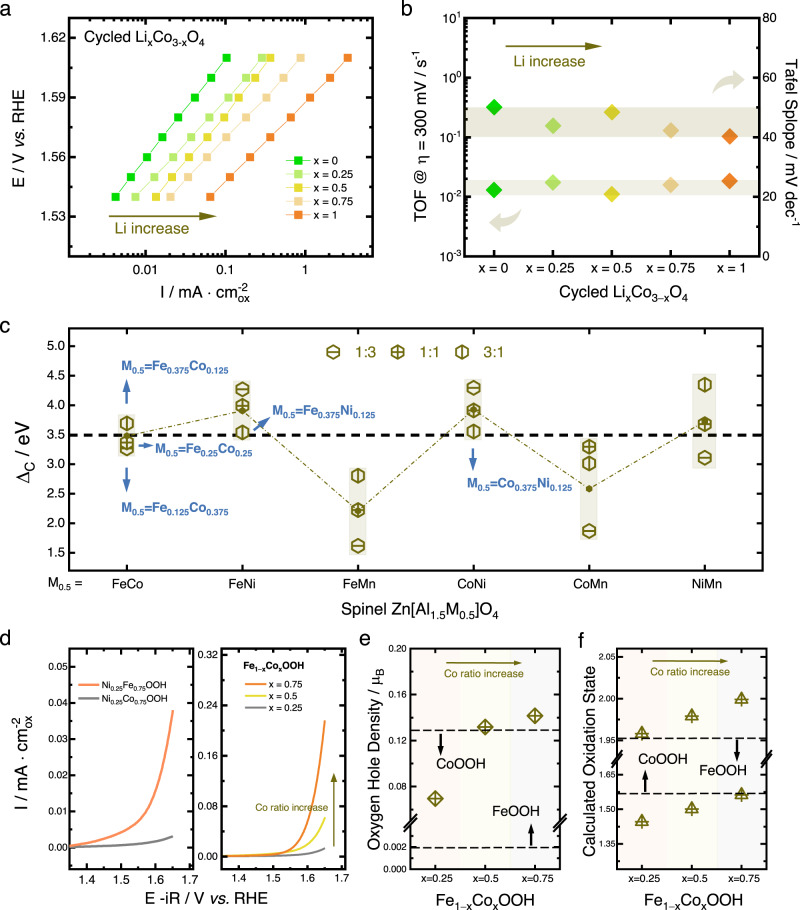
Investigation of the reconstructed oxyhydroxides from spinel pre-catalysts. **a** The Tafel plots of the cycled spinel Li_x_Co_3−x_O_4_. **b** The Tafel slope and turnover frequency (TOF) of cycled spinel Li_x_Co_3−x_O_4_. **c** The calculated structural polarity of spinel Zn[Al_1.5_M_0.5_]O_4_ (M = FeCo, FeNi, FeMn, CoNi, CoMn, and NiMn). For each bimetallic combination, three metal-metal ratios, i.e., 1:3, 1:1, and 3:1, are calculated. **d** The cyclic voltammetry (CV) curves of Ni_0.25_Fe_0.75_OOH, Ni_0.25_Co_0.75_OOH, and Fe_1–x_Co_x_OOH (*x* = 0.25, 0.5, and 0.75). The oxyhydroxides are in situ generated from spinel pre-catalyst Zn[Al_1.5_Ni_0.125_Fe_0.375_]O_4_, Zn[Al_1.5_Ni_0.125_Co_0.375_]O_4_, Zn[Al_1.5_Fe_0.375_Co_0.125_]O_4_, Zn[Al_1.5_Fe_0.25_Co_0.25_]O_4_, and Zn[Al_1.5_Fe_0.125_Co_0.375_]O_4_. The curves are capacitance-corrected by averaging the forward and backward sweeps. **e** The calculated oxygen hole density (μB) of Fe_1–x_Co_x_OOH (*x* = 0.25, 0.5, and 0.75). The oxygen hole density of FeOOH and CoOOH is also shown for reference. **f** The calculated oxidation state of cobalt and iron cations in Fe_1–x_Co_x_OOH (*x* = 0.25, 0.5, and 0.75). The dashed lines represent the oxidation state of iron cation in FeOOH and that of cobalt cation in CoOOH.

Although the profile of surface reconstruction on spinel oxides has been outlined, it remains ambiguous how to maximize the function of surface reconstruction towards optimal reaction performance. This necessitates the understanding of how surface reconstruction contributes to the measured OER activity and what the key factors dominating the activity of oxyhydroxides are. Generally, the activity enhancement of electrochemical reactions can be realized via two aspects, i.e., increasing the number of active sites or increasing the intrinsic activity of active sites^3^. To figure out how surface reconstruction contributes to the OER activity, electrochemical analysis of cycled Li_x_Co_3−x_O_4_ is performed. As shown in Fig. 4a, the measured OER activity significantly rises with the increase of amorphization degree, indicating the reaction performance is positively correlated to the degree of surface reconstruction. The intrinsic activities of the generated cobalt oxyhydroxides are compared in Fig. 4b. As shown, the Tafel slopes of the generated cobalt oxyhydroxides are quite close to each other, in which the maximum difference is less than 10 mv dec^−1^. This suggests all the cobalt oxyhydroxides show similar reaction mechanism and share the same rate-determining step^25^. The activity per cobalt site is evaluated by calculating the turnover frequency (TOF). As given in Fig. 4b, the TOF values of the five cobalt oxyhydroxides are within the range of 0.01 s^−1^ to 0.02 s^−1^. Considering the observation that TOF can range across many orders of magnitude, these values indicate the intrinsic activity of each cobalt site in the five oxyhydroxides is almost identical to each other^25^. Therefore, we can rationally deduce that, for pre-catalysts with the same metal cation, an increased reconstruction ability does not alter the intrinsic catalytic activity of the active sites in the in situ generated amorphous oxyhydroxide, but contributes to the enhanced activity by increasing the number of available active sites.

The demonstrated structure-reconstruction relationship also allows us to design spinel pre-catalysts with the same reconstruction ability, based on which we can in situ generate oxyhydroxides with the same amorphization degree to investigate the influence of cation type and ratio on the OER activity of oxyhydroxides. Specifically, the reconstruction ability of spinel Zn[Al_1.5_M_0.5_]O_4_ (M = FeCo, FeNi, FeMn, CoNi, CoMn, and NiMn) were screened to search for spinel pre-catalysts with the same reconstruction ability. The cations of iron, cobalt, nickel, and manganese were studied because they are earth-abundant and therefore exhibit cost advantage for potential commercial usage. The screening results are summarized in Fig. 4c. It can be seen that, in zinc aluminum spinel substrate, the introduction of octahedral nickel cation and manganese cation generally results in higher and lower reconstruction ability, respectively. The Δ_C_ value of 3.5 eV is chosen as a reference line to select spinel pre-catalysts with the same reconstruction ability. The Δ_C_ value of 3.5 eV is chosen because it guarantees a high-level reconstruction ability of spinel pre-catalyst and can incorporate sufficient screened samples with the same reconstruction ability to design control experiment for the catalytic investigations on the in situ generated oxyhydroxides. Specifically, spinel Zn[Al_1.5_Fe_0.125_Co_0.375_]O_4_, Zn[Al_1.5_Fe_0.25_Co_0.25_]O_4_, Zn[Al_1.5_Fe_0.375_Co_0.125_]O_4_, Zn[Al_1.5_Fe_0.375_Ni_0.125_]O_4_, and Zn[Al_1.5_Co_0.375_Ni_0.125_]O_4_ are selected to serve as pre-catalysts to generate the bimetallic oxyhydroxides. Subsequent experiments were conducted to synthesize the selected spinel pre-catalysts. The XRD characterization confirmed the spinel crystal of the prepared samples (Supplementary Figure 12). Electrochemical measurements were thereafter performed to in situ generate the corresponding amorphous oxyhydroxides. To guarantee a complete reconstruction, the measurements were conducted for at least 500 cycles until a stable electrochemical curve appears. The TEM images of the samples after reaction are shown in Supplementary Figure 13, which evidence the generation of amorphous layers and nearly the same amorphization degree. Besides, the point scan results (Supplementary Figure 14) confirmed the metal-metal ratio in the amorphous layers is about the same as that in the spinel pre-catalysts.

The electrochemical activity of the generated oxyhydroxides were analyzed and shown in Fig. 4d. The results demonstrate the activity of oxyhydroxides can be largely influenced by the cation type and cation ratio. The comparison between Ni_0.25_Fe_0.75_OOH and Ni_0.25_Co_0.75_OOH suggests that the reactivity of iron cation in oxyhydroxide is higher than that of cobalt cation, consistent with the conclusion reported in other reports^11,25^. The comparison between iron-cobalt oxyhydroxides with different Fe/Co ratio, however, shows a trend that the activity increases with the increase of cobalt ratio in oxyhydroxides. This is a phenomenon that inevitably occurs during OER, in which small amount of iron (normally less than 40%) can significantly raise the activity of cobalt-based oxyhydroxides^12,25–28^. There are two possible reasons. One is that the CoO_6_ units serve as a conductive and chemically stable host for iron species, which are intrinsically very active but are electrically insulating^25,28,29^. The other is that the formation of high-valent iron species (normally refers to Fe^4+^), serving either as direct catalytical centers or as redox activators for cobalt cations, is the key for the superior activity^27,28^. To identify the dominant factor for the iron-cobalt oxyhydroxides generated from spinel pre-catalysts, we further investigated the charge transport ability and cation oxidation state.

As elucidated elsewhere, the oxygen hole density is an effective index to estimate the charge transport ability within the transition metal oxides (TMOs), where a higher hole density suggests a higher charge transport ability^30–32^. The calculated oxygen hole density is presented in Fig. 4e. For FeOOH, only traceable hole density (0.002 μ_B_) is observed, indicating an insulating nature. When 25% cobalt cations are introduced (Fe_0.75_Co_0.25_OOH), the oxygen hole density dramatically increases and reaches 0.07 μ_B_, suggesting a significant increase of charge transport ability. When the cobalt ratio further increases to 50% (Fe_0.5_Co_0.5_OOH), the oxygen hole density undergoes another dramatic increase to reach 0.13 μ_B_. With further increasement of cobalt ratio to 75%, the oxygen hole density of Fe_0.25_Co_0.75_OOH is 0.14 μ_B_ and is close to that of Fe_0.5_Co_0.5_OOH, which implies that the charge transport ability within iron-cobalt oxyhydroxides may not undergo further increasement with additional incorporation of cobalt cations. Noting that the oxygen hole density on CoOOH is 0.129 μ_B_, the values of 0.13 μ_B_ on Fe_0.5_Co_0.5_OOH and 0.14 μ_B_ on Fe_0.25_Co_0.75_OOH indicate that their charge transport ability has already reaches the level at which it is no longer the bottleneck of the reaction. Therefore, we may conclude that the poor charge transport ability is the main catalytical drawback of iron-cobalt oxyhydroxide when the cobalt ratio is less than 50% (iron ratio higher than 50%).

The calculated cation oxidation states are shown in Fig. 4f. The two dashed lines represent the oxidation state of cobalt and iron in CoOOH and FeOOH, respectively. The trend evidences a gradual increase of iron oxidation state with the increase of cobalt ratio, which implies the presence of high-valent iron cations in low-iron-containing iron-cobalt oxyhydroxides. Considering the charge transport ability is no longer a catalytical drawback for iron-cobalt oxyhydroxides with cobalt ratio more than 50%, the activity enhancement with additional cobalt incorporation may attribute to the increased iron oxidation state that facilitates an in situ generation of high-valent iron cations under OER potential. Therefore, the design of highly active and cost-effective oxyhydroxides from spinel pre-catalysts should consider two aspects: (i) the high reconstruction ability that guarantees the availability of more active sites and (ii) the incorporation of conductive and high-valent iron cations that guarantees high intrinsic activity of the active sites.

### Potential of surface reconstruction ability of spinel oxides

**Fig. 5:**
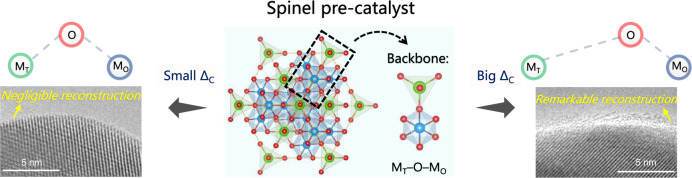
Schematic of the structure-reconstruction relationship for spinel pre-catalyst during water oxidation.

As demonstrated above, the structural origin of surface reconstruction on spinel oxides has been identified and a criterion evaluating the surface reconstruction ability of spinel oxides is unveiled (Fig. 5). To design spinel oxide with high reconstruction ability for the generation of more available active sites, strategies should be made to weaken the metal-oxygen covalency of MO_4_ and strengthen that of MO_6_. By contrast, if a spinel pre-catalyst is believed to hold remarkable intrinsic activity for OER, approaches are therefore needed to balance the metal-oxygen covalency between MO_4_ and MO_6_ to guarantee a stable in situ spinel crystal frame for long reaction durability.

**Fig. 6:**
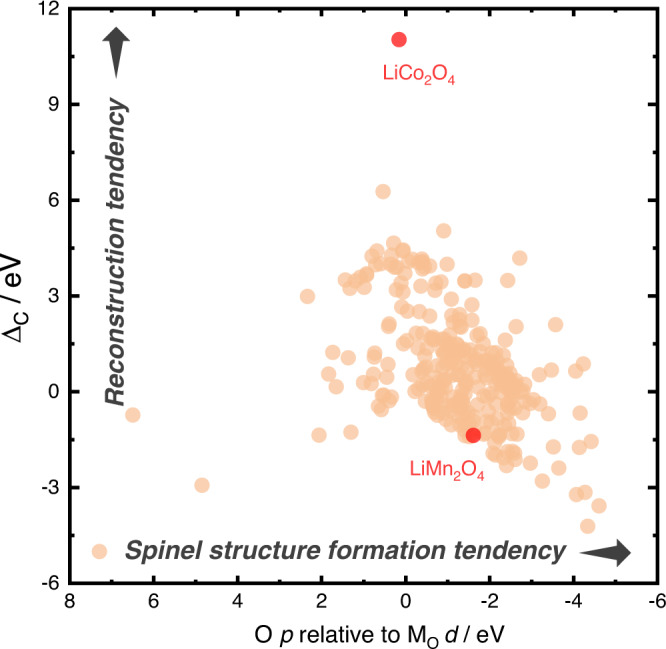
Surface reconstruction ability of various spinel oxides. High-throughput plot showing the reconstruction ability of various spinel oxides. The data points are taken from ref. ^17^ The *x*-axis is a parameter that can be used to evaluate the structure formation tendency of spinel oxides, which was proposed in ref. ^33^.

Employing the computational database established in our previous work^17^, a computational high-throughput figure can be plotted to evaluate the reconstruction ability of spinel oxides, which is shown in Fig. 6. The *x*-axis presents a parameter that can be used to evaluate the possibility of existence of spinel oxides, which is demonstrated in our previous study^33^. According to our findings, the value of O *p* states relative to M_O_ *d* states of phase-pure spinel oxides is normally below 1 eV^33^. It can be seen that most of the data points are located within the phase-pure region, ensuring the effectiveness of using this database to screen the properties of spinel oxides. As labeled, spinel LiCo_2_O_4_ exhibits tremendous potential for in situ surface reconstruction, indicating it can be used as an ideal pre-catalyst with high reconstruction ability to generate cobalt-oxyhydroxide-host for active cation (such as iron) substitution. Besides, it’s worth to note that the Δ_C_ value of spinel LiMn_2_O_4_ is only −1.373 eV, which indicates strong resistivity towards reconstruction. This explains why lithium cannot leach out from LiMn_2_O_4_ lattice and therefore the spinel structure remains almost unchanged during OER. The overall Δ_C_ range of spinel oxides is from −6 eV to 12 eV, which demonstrates a huge structural flexibility to manipulate the reconstruction degree. Therefore, we can conclude that spinel oxides are a big family of transition metal oxides with high flexibility to serve as pre-catalysts for electrochemical water oxidation.

In summary, combining computational and experimental analysis, we have pinpointed and demonstrated the structure-reconstruction relationship of surface reconstruction on spinel oxides. The metal-oxygen covalency polarity of the M_T_–O–M_O_ backbone is proved to be the structural origin that triggers surface reconstruction, in which higher polarity corresponds to greater degree of reconstruction. With the established structure-reconstruction relationship, the reconstruction ability of spinel pre-catalysts can be precisely predicted and subtly manipulated. Besides, by conducting mechanistic study on a series of oxyhydroxides generated from spinel pre-catalysts, we have showcased how surface reconstruction contributes to OER activity and how activity of the generated oxyhydroxides evolves with the change of cation type and cation ratio. Our work unveils the key fundamentals of surface reconstruction on spinel oxides and provides firm theoretical foundations for the spinel pre-catalyst design. The research scenario for studying the surface reconstruction may also be extended to other types of TMOs.

## Methods

### Density functional theory (DFT) calculations

The DFT calculations were performed by the Vienna Ab-initio Simulation Package^34,35^ (VASP), employing the Projected Augmented Wave^36^ (PAW) method. The exchange and correlation effects were described by using the Perdew-Burke-Ernzerhof^37^ (PBE) functional. The cutoff energy was set to be 450 eV in all the cases. The GGA + U calculations were performed using the model proposed by Dudarev et al^38^. The effective Hubbard U values were set to be 4.5 eV, 4.7 eV, 3.3 eV, 4 eV, 6.4 eV, and 4 eV for tetrahedral Co, tetrahedral Zn, octahedral Co, octahedral Fe, octahedral Ni, and octahedral Mn, respectively. The Monkhorst-Pack grids were set to be 6 × 6 × 6 and 9 × 9 × 9 for performing the structural optimization and density of states calculations, respectively. The conventional standard cell of spinel oxide is employed in this work, in which 8 tetrahedral cations, 16 octahedral cations and 32 oxygen anions are contained. During the model construction, all the lithium cations in the lithium-containing spinels are placed in the tetrahedral sites, as both theoretical^39^ and experimental^40,41^ proofs have demonstrated the energetical preference of lithium in tetrahedral sites. The different placement of cobalt and lithium cations in tetrahedral sites does not affect the derived band center of ions since no M_T_–O–M_T_ bond exists in spinel structure.

The vacancy formation energy was calculated as the energy difference between the vacant spinel together with an oxygen atom and the intact spinel structure, which was defined as

2 $${E}_{{vac}-O}={E}_{{vacant}-{bulk}}+{E}_{{oxygen}}-{E}_{{intact}-{bulk}}.$$

where ${E}_{{vacant}-{bulk}}$, ${E}_{{oxygen}}$, and ${E}_{{intact}-{bulk}}$ represent the electronic energy of the spinel with one bulk oxygen vacancy, the chemical potential of one oxygen atom referenced to gaseous oxygen molecule, and the electronic energy of the intact spinel, respectively^42^. Under this definition, a lower value of bulk oxygen formation energy suggests an easier case for generating an oxygen vacancy.

The surface transformation energy from spinel to layered oxide was calculated as the energy difference (per surface area) between the reconstructed species and the intact spinel surface, which was defined as

3 $${E}_{{spinel}\,{to}\,{CoOO}}=\frac{{E}_{{spinel}-{slab}}-{n}_{1}{E}_{{CoOO}}-{n}_{2}{Co}-{n}_{3}{Li}}{{S}^{2}}.$$

where ${E}_{{spinel}-{slab}}$, ${E}_{{CoOO}}$, ${n}_{1}$, ${n}_{2}$, ${n}_{3}$, and *S* represent the electronic energy of the spinel slab model, the layered CoOO species, the number of reconstructed CoOO species, the number of cobalt cations in the tetrahedral sites of spinel slab, the number of lithium cations in the tetrahedral sites, and the surface area of the spinel slab, respectively. Under this definition, a lower value of surface transformation energy suggests an easier transformation from spinel structure to layered structure.

### Synthesis of the spinel pre-catalysts

The spinel pre-catalysts were synthesized by a modified conventional sol-gel combustion method. Metal acetates Co(CH_3_COO)_2_·4H_2_O (99.999% trace metals basis, Sigma-Aldrich), Zn(CH_3_COO)_2_·2H_2_O, Mn(CH_3_COO)_2_·4H_2_O, Ni(CH_3_COO)_2_·4H_2_O, Fe(NO_3_)_3_·9H_2_O (Sigma-Aldrich), and LiNO_3_ (Sigma-Aldrich) with a specific molar ratio were first mixed in dilyted nitric acid solution through vigorous stirring. Citric acid (ACS reagent, ≥99.5%, Sigma-Aldrich) acting as chelating agent, was the added in the mixture. Under the condition of constant stirring at 90 °C for several hours, the mixture solution converts into viscous gel. The gel was then decomposed in air at 170 °C (with a heating rate of 10 °C min^−1^ to 170 °C) for 12 hours to remove the remaining water and ground thoroughly. After applying a further heat treatment in air at 400 °C for six hours, phase pure spinel oxide powders were obtained.

### Procedures of electrochemical analysis

The OER analysis was operated in a three-electrode cell with a working electrode of glassy carbon flake (effective electrode area: 0.196 cm^2^), a counter electrode of platinum foil, and a Hg/HgO reference electrode filled with 1 M KOH solution. The 1 M KOH solution was prepared by using electronic grade KOH (purity 99.999%, Aladdin), which excludes the Fe contamination issue^43^. The catalyst electrode was fabricated by the recipe drop-castes method. The catalysts were mixed with acetylene black (AB) at a mass ration of 5:1, then dispersed in isopropanol/water (v/v = 1:4) solvent followed by the addition of Na^+^-exchanged Nafion as the binder. The mixtures were ultrasonicated for 30 min to reach homogeneous ink. The concentration of oxides in ink is 5 mg/ml, and AB is 1 mg/ml. Before drop-casting, the glassy carbon electrodes were polished to a mirror with α-Al_2_O_3_ (50 nm) and washed by IPA and water to clean up completely. Finally, the as-prepared ink (10 ul) was dropped onto glassy carbon electrodes to reach a loading mass of 50 μg_ox_ cm^−2^ and the electrodes were dried overnight at room temperature. Cyclic voltammograms (CVs), linear sweep voltammetry (LSV) and chronoamperometry (CA) were performed in O_2_-saturated 1 M KOH by using Bio-logic SP 150 potentiostat. The potential range was set from 0.90 V to 1.62 V (*vs*. RHE). The scan rate was kept at 10 mV s^–1^. All potentials were converted to RHE scale with iR correction according to

4 $$E\left({vs}.{RHE}\right)=E\left({vs}.\,{Hg}/{HgO}\right)+0.059*{pH}+0.098.$$

The pH value of the electrolyte was obtained from pH meter measurement by using Orion StarTM A121 portable pH meter (Thermo Fisher Scientific), from which pH values of 14.00 ± 0.05 were measured in this work. The internal resistance was determined right after the CV measurements by electrochemical impedance spectroscopy (EIS). The EIS measurement was conducted by collecting the AC impedance spectra from 200 kHz to 1 Hz with a voltage perturbation of 10 mV versus a constant potential. The internal resistance was the real part of the impedance where the imaginary part of the impedance was zero in the Nyquist plot. The measured internal resistance is 5.00 ± 0.05 Ω in this work. The OER polarization curves were then corrected to obtain the iR-free potential (E -iR) of the working electrode.

### Turnover frequency (TOF) calculations

In this work, the TOFs were calculated via the redox method, which is commonly employed in the system of oxyhydroxides. Specifically, the TOF calculation is based on the following equation:

5 $${TOF}=\frac{{I}_{{ox}}\times {A}_{{ox}}}{4\times e\times N}=\frac{E}{4\times e\times N}.$$

where ${I}_{{ox}}$, ${A}_{{ox}}$, *E*, *e*, and *N* represent the BET normalized current density at the overpotential of 300 mV, the oxide surface area measured by BET, the measured current on the electrode, the charge of a single electron, and the number of cobalt atoms in the electrode. The number of cobalt atoms (N) is estimated by the integration of Co redox peak at ≈1.15 V vs. RHE (assuming a one-electron process of Co^2+^/Co^3+^).

### Derivation of surface reconstruction degree (D_r_)

The surface reconstruction degree was evaluated on the basis that the loaded catalysts can be considered as a sphere model. With the measurement of Brunauer-Emmett-Teller (BET), the total surface area of the loaded pre-catalyst can be obtained by ${m}_{{loading}}{\cdot BET}$. So the following equation can be obtained

6 $${m}_{{loading}}{{\cdot }}{BET}=4\pi {({R}_{{loading}})}^{2}.$$

Based on above equation, the radius of the loaded pre-catalyst (${R}_{{load}}$) can be obtained as

7 $${R}_{{loading}}=\sqrt{\frac{{m}_{{loading}}{{\cdot }}{BET}}{4\pi }}.$$

The volume of the loaded pre-catalyst can therefore be evaluated as

8 $${V}_{{loading}}=\frac{4}{3}{{\cdot }}\pi {{\cdot }}{\left(\sqrt{\frac{{m}_{{loading}}{{\cdot }}{BET}}{4\pi }}\right)}^{3}.$$

The reconstructed part can be evaluated by the solvated lithium cations in the electrolyte. By conducting inductively coupled plasma (ICP) analysis, the content of the solvated lithium cations (${c}_{{Li}}$, with the unit of *g*) can be obtained. The mole of lithium cations can then be obtained as $\frac{{c}_{{Li}}}{6.94}$, where 6.94 (*g mol*^*−1*^) is the molar mass of lithium. The number of lithium cations in the solution can be evaluated as $\frac{{c}_{{Li}}\cdot {N}_{A}}{6.94}$, where ${N}_{A}$ is the Avogadro constant. The number of the solvated spinel molecule can be evaluated as $\frac{{c}_{{Li}}\cdot {N}_{A}}{6.94{\cdot x}}$, where *x* is the stochiometric number in spinel Li_x_Co_3−x_O_4_. The reconstructed volume of spinel pre-catalyst can therefore be evaluated as

9 $${V}_{{reconstruction}}=\frac{{c}_{{Li}}{{\cdot }}{N}_{A}}{6.94{{\cdot }}x}{{\cdot }}{V}_{{mol}}.$$

where ${V}_{{mol}}$ is the volume of one spinel molecule obtained from DFT calculations. Based on above derivations, the reconstruction degree (D_r_) can be evaluated as

10 $${D}_{r}=\frac{{V}_{{reconstruction}}}{{V}_{{loading}}}=\frac{\frac{{c}_{{Li}}{{\cdot }}{N}_{A}}{6.94{{\cdot }}x}{{\cdot }}{V}_{{mol}}}{\frac{4}{3}{{\cdot }}\pi {{\cdot }}{\left(\sqrt{\frac{{m}_{{loading}}{{\cdot }}{BET}}{4\pi }}\right)}^{3}}.$$

### Materials characterization

The X-ray diffraction (XRD) of spinel oxides were carried out on Bruker D8 diffractometer at a scanning rate of 2° min^−1^, under Cu-Kα radiation (λ = 1.5418 Å). The X-ray photoelectron spectroscopy (XPS) measurements were performed using PHI-5400 equipment with Al Kα beam source (250 W) and a position-sensitive detector (PSD) was used to determine the surface composition of the materials. Fourier transform infrared spectroscopy (FTIR) data were recorded using FTIR PerkinElmer Spectrum. The inductively coupled plasma-optical emission spectrometry (ICP-OES) characterizations were performed using Agilent 720 with a detection limit of 0.02 ppm. The sample powders were weighed (accuracy 0.1 mg) and put in a dry beaker. Then 4 mL of aqua regia solution was added into the beaker and heated at 80 °C until the powders were fully dissolved. The solution was allowed to cool naturally to room temperature in fume hood before being transferred into a 25 mL volumetric flask. Deionized (DI) water was added to dilute the solution to 25 mL and was shaked thoroughly for ICP-OES testing. The X-ray absorption near edge spectroscopy (XANES) and extended X-ray absorption fine structure (EXAFS) experiments were conducted in transmission mode at beamline XAFCA of the Singapore Synchrotron Light Source^44^. The data reduction and data analysis were performed with the Athena, Artemis and IFEFFIT software packages. The EXAFS data were extracted from the measured absorption spectra using standard procedures. A cubic-spline-fit procedure was used to determine the post-edge background with a subsequent subtraction. Normalization was performed by dividing the data by the height of the absorption edge at 50 eV. The results of Brunauer-Emmett-Teller (BET) measurements are summarized in Supplementary Figure 2 and Supplementary Table 3.

## Supplementary information

Supplementary Information

## Data Availability

The data supporting the findings of this study are available within the article and its Supplementary Information. Additional data are available from the corresponding author upon reasonable request.

## References

[1] Staffell I (2019). The role of hydrogen and fuel cells in the global energy system. Energy Environ. Sci..

[2] Stamenkovic VR, Strmcnik D, Lopes PP, Markovic NM (2017). Energy and fuels from electrochemical interfaces. Nat. Mater..

[3] Seh ZW (2017). Combining theory and experiment in electrocatalysis: Insights into materials design. Science.

[4] Nørskov JK, Abild-Pedersen F, Studt F, Bligaard T (2011). Density functional theory in surface chemistry and catalysis. Proc. Natl Acad. Sci. USA.

[5] Nørskov JK, Bligaard T, Rossmeisl J, Christensen CH (2009). Towards the computational design of solid catalysts. Nat. Chem..

[6] Seitz LC (2016). A highly active and stable IrO x/SrIrO3 catalyst for the oxygen evolution reaction. Science.

[7] Suntivich J, May KJ, Gasteiger HA, Goodenough JB, Shao-Horn Y (2011). A perovskite oxide optimized for oxygen evolution catalysis from molecular orbital principles. Science.

[8] Gao L, Cui X, Sewell CD, Li J, Lin Z (2021). Recent advances in activating surface reconstruction for the high-efficiency oxygen evolution reaction. Chem. Soc. Rev..

[9] Liu X (2021). Comprehensive understandings into complete reconstruction of precatalysts: synthesis, applications, and characterizations. Adv. Mater..

[10] Fabbri E (2017). Dynamic surface self-reconstruction is the key of highly active perovskite nano-electrocatalysts for water splitting. Nat. Mater..

[11] Burke MS, Enman LJ, Batchellor AS, Zou S, Boettcher SW (2015). Oxygen evolution reaction electrocatalysis on transition metal oxides and (oxy) hydroxides: activity trends and design principles. Chem. Mater..

[12] Dionigi F (2021). Intrinsic electrocatalytic activity for oxygen evolution of crystalline 3d‐transition metal layered double hydroxides. Angew. Chem..

[13] Chen Y (2021). Lattice site–dependent metal leaching in perovskites toward a honeycomb-like water oxidation catalyst. Sci. Adv..

[14] Wang J (2021). Redirecting dynamic surface restructuring of a layered transition metal oxide catalyst for superior water oxidation. Nat. Catal..

[15] Köhler L, Abrishami MEbrahimizadeh, Roddatis V, Geppert J, Risch M (2017). Mechanistic parameters of electrocatalytic water oxidation on LiMn2O4 in comparison to natural photosynthesis. ChemSusChem.

[16] Wei C (2017). Cations in octahedral sites: a descriptor for oxygen electrocatalysis on transition‐metal spinels. Adv. Mater..

[17] Sun Y (2020). Covalency competition dominates the water oxidation structure–activity relationship on spinel oxides. Nat. Catal..

[18] Tronel F (2006). New spinel cobalt oxides, potential conductive additives for the positive electrode of Ni−MH batteries. Chem. Mater..

[19] Xu L (2016). Plasma‐engraved Co3O4 nanosheets with oxygen vacancies and high surface area for the oxygen evolution reaction. Angew. Chem..

[20] Barreca D (2001). Composition and microstructure of cobalt oxide thin films obtained from a novel cobalt (II) precursor by chemical vapor deposition. Chem. Mater..

[21] Yang W-D, Hsieh C-Y, Chuang H-J, Chen Y-S (2010). Preparation and characterization of nanometric-sized LiCoO2 cathode materials for lithium batteries by a novel sol–gel method. Ceram. Int..

[22] Mouhib Y, Belaiche M, Ferdi CA, Lacham M, Elacham A (2020). New technique for elaboration and characterization of a high voltage spinel LiCo 2 O 4 cathode and theoretical investigation. N. J. Chem..

[23] Wei C (2019). Approaches for measuring the surface areas of metal oxide electrocatalysts for determining their intrinsic electrocatalytic activity. Chem. Soc. Rev..

[24] Zhou Y (2018). Enlarged Co O covalency in octahedral sites leading to highly efficient spinel oxides for oxygen evolution reaction. Adv. Mater..

[25] Burke MS, Kast MG, Trotochaud L, Smith AM, Boettcher SW (2015). Cobalt–iron (oxy) hydroxide oxygen evolution electrocatalysts: the role of structure and composition on activity, stability, and mechanism. J. Am. Chem. Soc..

[26] Enman LJ (2018). Operando x‐ray absorption spectroscopy shows iron oxidation is concurrent with oxygen evolution in cobalt–iron (oxy) hydroxide electrocatalysts. Angew. Chem..

[27] Li N, Hadt RG, Hayes D, Chen LX, Nocera DG (2021). Detection of high-valent iron species in alloyed oxidic cobaltates for catalysing the oxygen evolution reaction. Nat. Commun..

[28] Lee, S., Moysiadou, A., Chu, Y.-C., Chen, H. M. & Hu, X. Tracking high-valent surface iron species in the oxygen evolution reaction on cobalt iron (oxy) hydroxides. *Energy Environ. Sci.*10.1039/D1EE02999A (2022).

[29] Zhang T, Nellist MR, Enman LJ, Xiang J, Boettcher SW (2019). Modes of Fe incorporation in Co–Fe (Oxy) hydroxide oxygen evolution electrocatalysts. ChemSusChem.

[30] Sun Y (2020). Spin‐related electron transfer and orbital interactions in oxygen electrocatalysis. Adv. Mater..

[31] Zhang L, Cheruvathur A, Biz C, Fianchini M, Gracia J (2019). Ferromagnetic ligand holes in cobalt perovskite electrocatalysts as an essential factor for high activity towards oxygen evolution. Phys. Chem. Chem. Phys..

[32] Ju H, Sohn H-C, Krishnan KM (1997). Evidence for O 2 p hole-driven conductivity in La 1−x Sr x MnO 3 (0≤ x≤ 0.7) and La 0.7 Sr 0.3 MnO z thin films. Phys. Rev. Lett..

[33] Duan Y (2019). Mastering surface reconstruction of metastable spinel oxides for better water oxidation. Adv. Mater..

[34] Kresse G, Hafner J (1994). Ab initio molecular-dynamics simulation of the liquid-metal–amorphous-semiconductor transition in germanium. Phys. Rev. B.

[35] Kresse G, Furthmüller J (1996). Efficient iterative schemes for ab initio total-energy calculations using a plane-wave basis set. Phys. Rev. B.

[36] Blöchl PE (1994). Projector augmented-wave method. Phys. Rev. B.

[37] Perdew JP, Burke K, Ernzerhof M (1996). Generalized gradient approximation made simple. Phys. Rev. Lett..

[38] Dudarev SL, Botton GA, Savrasov SY, Humphreys C, Sutton AP (1998). Electron-energy-loss spectra and the structural stability of nickel oxide: An LSDA+ U study. Phys. Rev. B.

[39] Van der Ven A, Ceder G (1999). Electrochemical properties of spinel Li x CoO 2: A first-principles investigation. Phys. Rev. B.

[40] Sun S (2019). Shifting oxygen charge towards octahedral metal: a way to promote water oxidation on cobalt spinel oxides. Angew. Chem..

[41] Choi S, Manthiram A (2002). Chemical synthesis and properties of spinel Li1−xCo2O4−δ. J. Solid State Chem..

[42] Lee Y-L, Kleis J, Rossmeisl J, Shao-Horn Y, Morgan D (2011). Prediction of solid oxide fuel cell cathode activity with first-principles descriptors. Energy Environ. Sci..

[43] Ren X (2020). Constructing an adaptive heterojunction as a highly active catalyst for the oxygen evolution reaction. Adv. Mater..

[44] Du Y (2015). XAFCA: a new XAFS beamline for catalysis research. J. Synchrotron Radiat..

