# Supplementary material for: Navigating surface reconstruction of spinel oxides for electrochemical water oxidation
Source: Nat Commun. 2023 Apr 28;14:2467. doi: 10.1038/s41467-023-38017-3 (PMC10147629; doi:10.1038/s41467-023-38017-3)
Supplement: Supplementary file 1 — Supplementary Information [file 41467_2023_38017_MOESM1_ESM.pdf]

Supplementary Information

Navigating Surface Reconstruction of Spinel Oxides for Electrochemical Water Oxidation

Yuanmiao Sun[1,2,12], Jiarui Wang[3,4,12], Shibo Xi[5,12], Jingjing Shen[3], Songzhu Luo[3], Jingjie Ge[6], Shengnan Sun[7], Yubo Chen[3], John V. Hanna[3,8], Shuzhou Li[3], Xin Wang[9], Zhichuan J. Xu[3,10,11]*

[1]Faculty of Materials Science and Energy Engineering/Institute of Technology for Carbon Neutrality, Shenzhen Institute of Advanced Technology, Chinese Academy of Sciences, Shenzhen 518055, P. R. China.

[2]Shenzhen Key Laboratory of Energy Materials for Carbon Neutrality, Shenzhen Institute of Advanced Technology, Chinese Academy of Sciences, Shenzhen 518055, P. R. China.

[3]School of Materials Science and Engineering, Nanyang Technological University, 50 Nanyang Avenue, Singapore 639798, Singapore.

[4]Singapore-HUJ Alliance for Research and Enterprise (SHARE), NEW-CREATE Phase II, Campus for Research Excellence and Technological Enterprise (CREATE), Singapore 138602, Singapore.

[5]Institute of Chemical and Engineering Science, Agency for Science Technology and Research (A*Star), Singapore 627833, Singapore.

[6]Department of Chemical and Biological Engineering, HKUST Jockey Club Institute for Advanced Study, Energy Institute, The Hong Kong University of Science and Technology, Clear Water Bay, Kowloon, Hong Kong, China.

[7]Institute of Materials Research and Engineering (IMRE), Agency for Science, Technology and Research (A*Star), 2 Fusionopolis Way, Singapore 138634, Singapore.

[8]Department of Physics, University of Warwick, Coventry, UK.

[9]Department of Chemistry, City University of Hong Kong, Hong Kong, China.

[10]Energy Research Institute @ Nanyang Technological University, ERI@N, Interdisciplinary Graduate School, Nanyang Technological University, 50 Nanyang Avenue, Singapore 639798, Singapore.

[11]Center for Advanced Catalysis Science and Technology, Nanyang Technological University, 50 Nanyang Avenue, Singapore 639798, Singapore.

[12]These authors contributed equally to this work.

*Corresponding author. Email: xuzc@ntu.edu.sg

Supplementary Table 1 | The inductively coupled plasma optical emission spectrometry (ICP-OES) results of the as-prepared spinel $Li_xCo_{3-x}O_4$.

Sample	Weight (g)	Digested Solution Colime (mL)	Elements	Concentration in Solution (ppm, mg/L)	Dilution Factor	Concentration in Powder (ppm, mg/Kg)	Weigh Ratio (wt%)	ICP composition
x=0.25	0.0427	10	Co	2.91	1000	681324.1	68.13%	$Li_{0.26}Co_{2.75}$
			Li	1.59	20	7466.1	0.75%	
x=0.5	0.0485	10	Co	3.32	1000	683746.4	68.37%	$Li_{0.47}Co_{2.5}$
			Li	3.66	20	15083.0	1.51%	
x=0.75	0.0429	10	Co	2.92	1000	679690.0	67.97%	$Li_{0.76}Co_{2.25}$
			Li	5.86	20	27301.7	2.73%	
x=1	0.0451	10	Co	3.01	1000	668095.3	66.81%	$Li_{0.94}Co_2$
			Li	8.36	20	37093.3	3.71%	

Supplementary Table 2 | The quantitative atomic ratio of Co^{2+}/Co^{3+} in spinel $Li_xCo_{3-x}O_4$ generated from X-ray photoelectron spectroscopy (XPS).

Co^{2+}/Co^{3+} ratio	x=0	x=0.25	x=0.5	x=0.75	x=1
Co $2p_{1/2}$	1.35	1.07	1.01	0.96	0.91
Co $2p_{3/2}$	1.41	1.10	0.81	0.78	0.77

Supplementary Table 3 | Summary of the Brunauer-Emmett-Teller (BET) surface area and average particle size of spinel $Li_xCo_{3-x}O_4$.

	x=0	x=0.25	x=0.5	x=0.75	x=1
BET surface area (m^2/g)	12.6950	4.3849	2.5754	1.2184	0.3820
Average particle size (nm)	472.6286	1368.3205	2329.7784	4924.5089	15705.5660

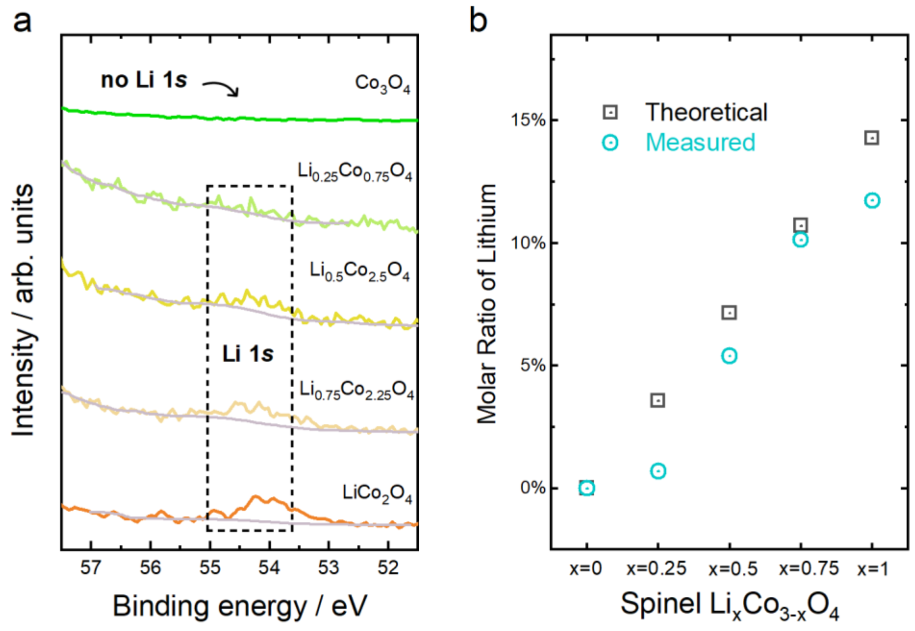

Supplementary Figure 1 | a. The recorded Li 1*s* X-ray photoelectron spectroscopy (XPS) of the pristine samples. **b.** The lithium molar ratio of the pristine samples. The black dots represent the theoretical lithium molar ratio calculated from the stoichiometric composition of spinel $Li_xCo_{3-x}O_4$. The cyan dots represent the measured lithium molar ratio that derived from Li 1*s* XPS results.

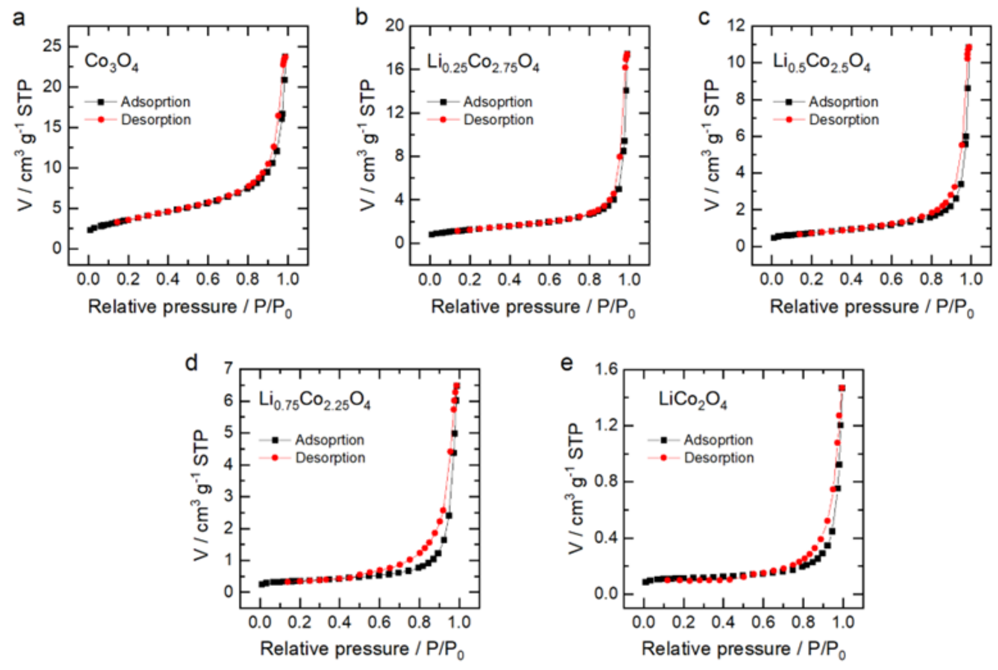

Supplementary Figure 2 | The Brunauer-Emmett-Teller (BET) measurements of the prepared spinel a) Co_3O_4, b) $Li_{0.25}Co_{2.75}O_4$, c) $Li_{0.5}Co_{2.5}O_4$, d) $Li_{0.75}Co_{2.25}O_4$, and e) $LiCo_2O_4$.

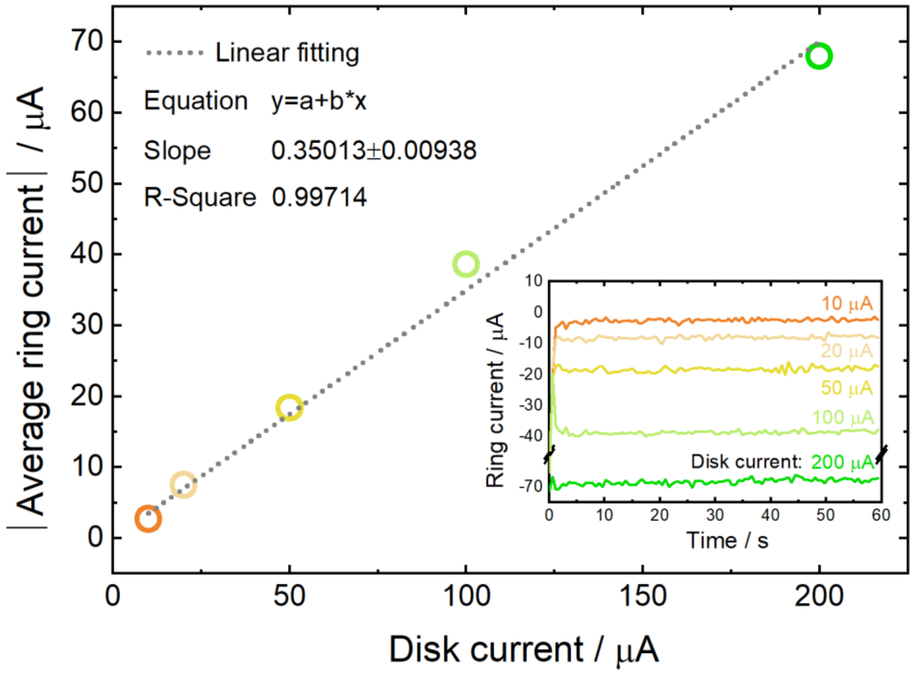

Supplementary Figure 3 | Linear fitting of the disk currents and the average ring currents (absolute values). The insert shows the measured ring currents at different disk currents.

The method for measuring the current efficiency of oxygen production was detailed in *Nat. Commun. 2022, 13, 5510*, in which a rotating ring-disk electrode (RRDE) voltammogram is measured and the experimental collection efficiency is calculated based on the measured disk and ring current during the redox of ferricyanide/ferrocyanide in potassium ferricyanide ($K_3Fe(CN)_6$). The evolved oxygen from the disk electrode (loaded with spinel $LiCo_2O_4$) is detected with the Pt-ring electrode, on which a four-electron oxygen reduction reaction occurs. As exhibited, chronopotentiometry is measured with the disk currents change from 10 µA to 200 µA. The corresponding ring currents are collected with a duration of 60 s. The average ring current is calculated by averaging the measured ring currents from 10 s to 60 s. By linearly fitting the measured disk currents and the average ring currents (absolute values), the Faradic efficiency of oxygen production can be estimated via the following equation:

$$F = \frac{I_{OER}}{I_{disk}} = \frac{(I_{ring} - i_a)/N_{exp}}{I_{disk}} = \frac{(I_{ring} - i_a)/I_{disk}}{N_{exp}} = \frac{b_{slope}}{N_{exp}}$$

where I_{OER} is the current from oxygen evolution reaction, I_{disk} is the measured disk current, I_{ring} is the measured ring current (absolute value), i_a is the background current in the ring disk, N_{exp} is the experimental collection efficiency, and b_{slope} is the slope of the linear fitting. Based on above equation, a Faradaic efficiency of 95.4 ± 2.5% is obtained for the disk electrode loaded with spinel $LiCo_2O_4$. Therefore, we conclude that the measured current mainly comes from oxygen evolution reaction.

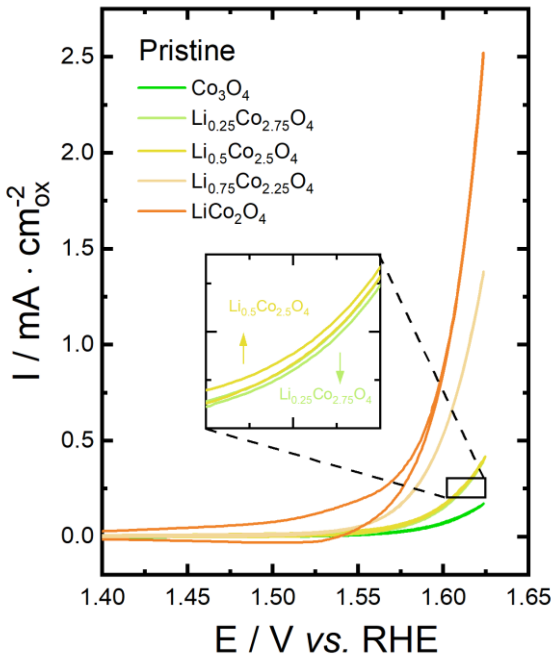

Supplementary Figure 4 | The non-iR compensated cyclic voltammetry (CV) of the pristine spinel $Li_xCo_{3-x}O_4$.

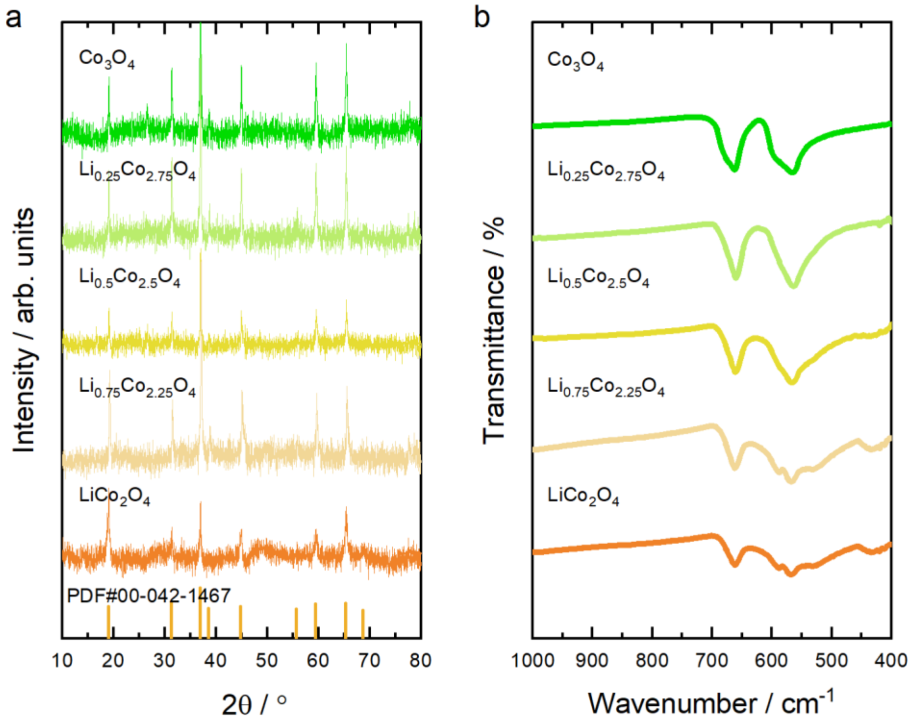

Supplementary Figure 5 | a. The X-ray Diffraction (XRD) patterns of the cycled spinel $Li_xCo_{3-x}O_4$. **b.** The Fourier Transform Infrared (FTIR) spectra of the cycled spinel $Li_xCo_{3-x}O_4$.

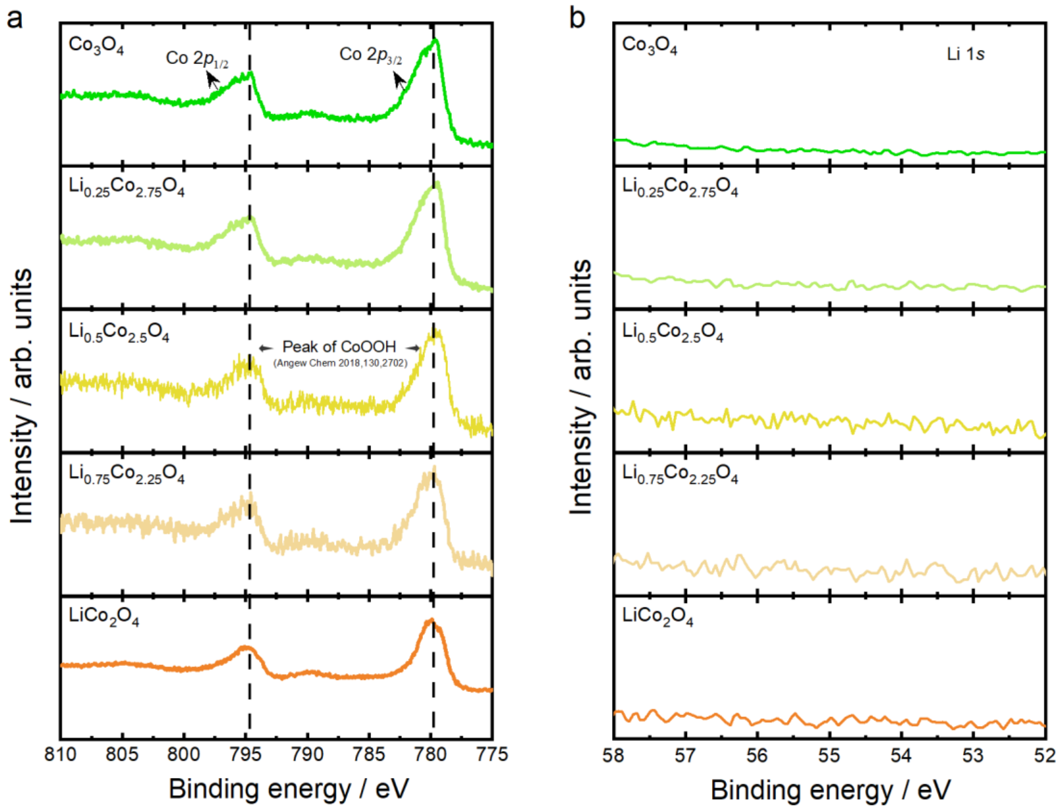

Supplementary Figure 6 | The recorded Co 2*p* (a) and Li 1*s* (b) X-ray photoelectron spectroscopy (XPS) of the fully cycled samples.

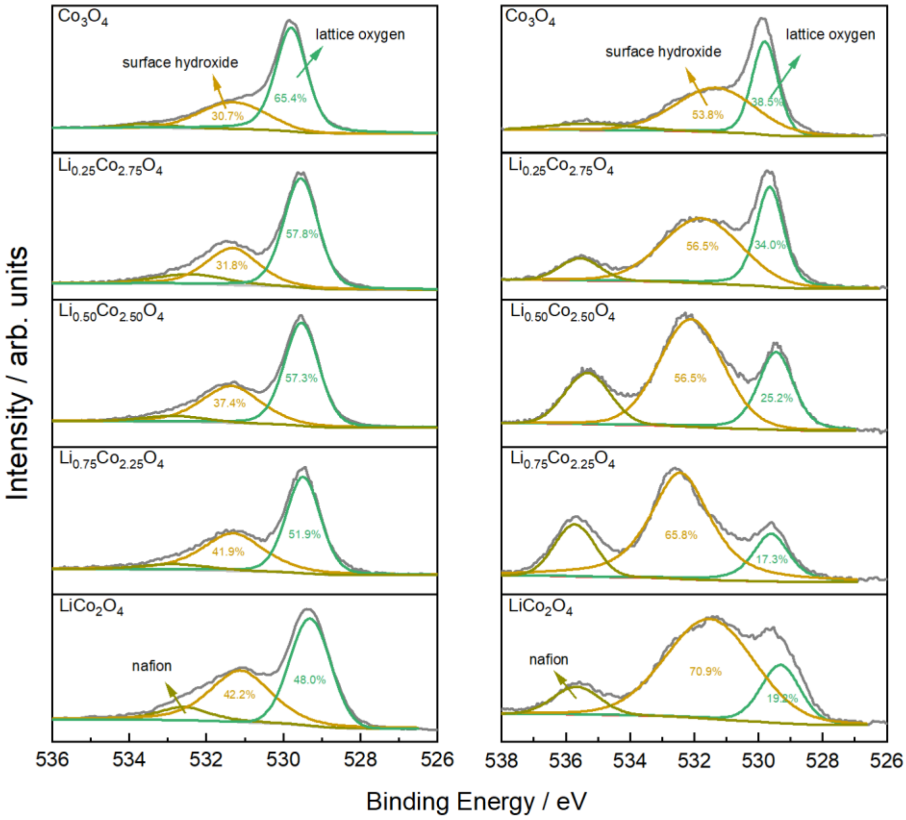

Supplementary Figure 7 | The recorded O 1*s* X-ray photoelectron spectroscopy (XPS) of the pristine and cycled samples. The left panel shows the results of pristine samples while the right shows those of the cycled ones.

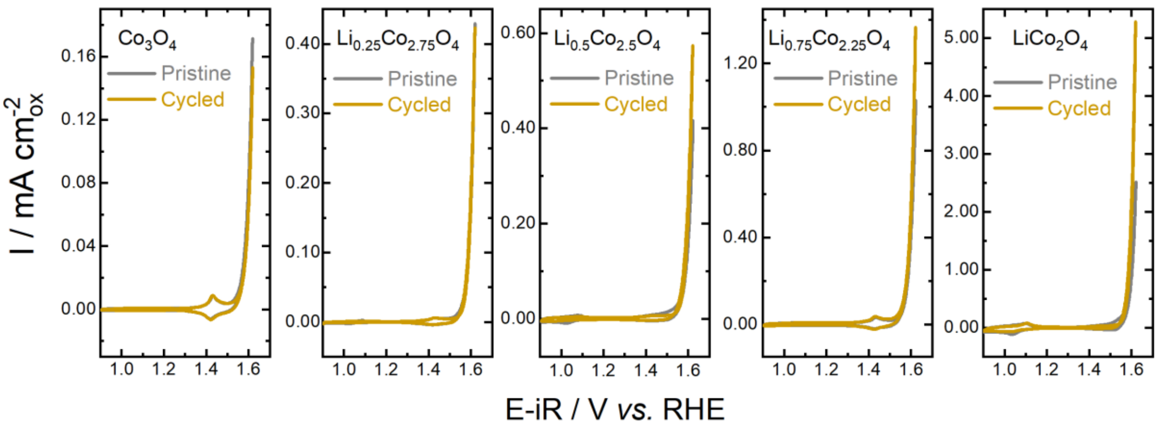

Supplementary Figure 8 | The cyclic voltammetry (CV) curves of all the pristine and cycled spinel $Li_xCo_{3-x}O_4$.

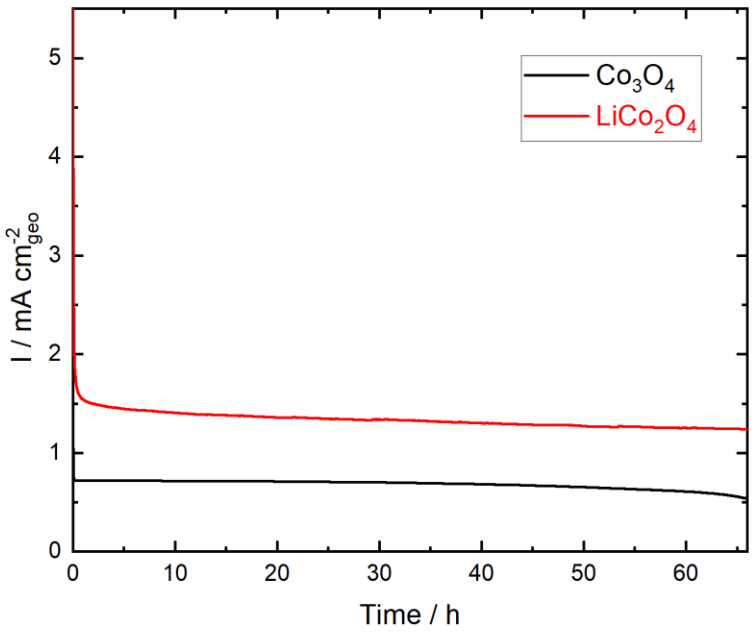

Supplementary Figure 9 | Chronoamperometry tests of spinel Co_3O_4 and $LiCo_2O_4$. The potential is controlled at 1.65 V vs. RHE.

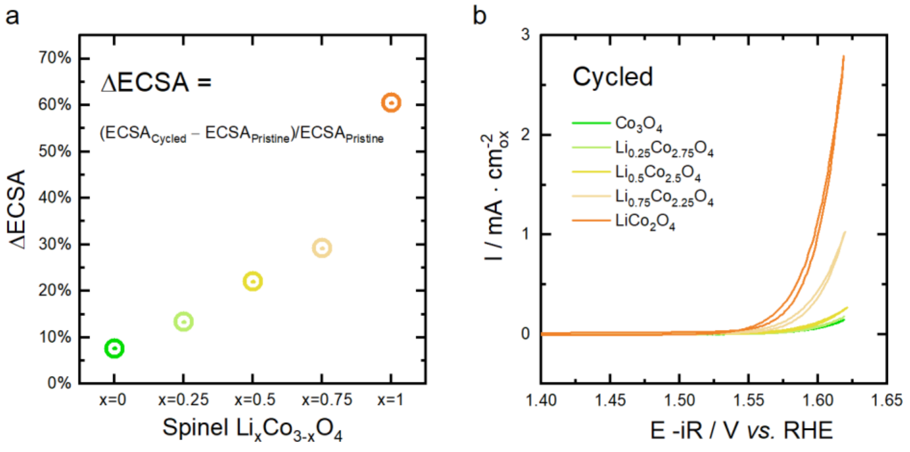

Supplementary Figure 10 | a. The increase of electrochemically active surface area (ECSA) between the pristine and cycled samples. **b.** The cyclic voltammetry (CV) curves of the cycled spinel $Li_xCo_{3-x}O_4$. The current densities are normalized by the measured ECSA of the cycled samples.

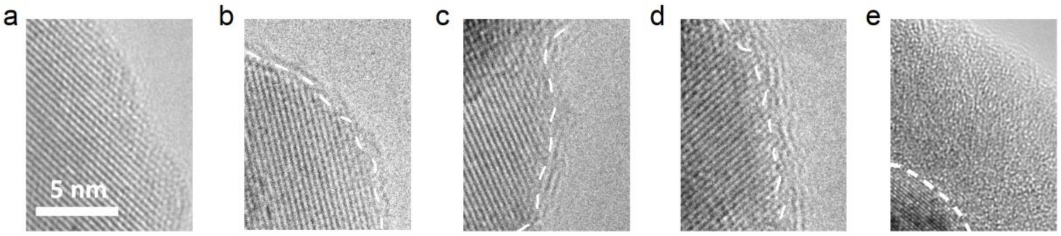

Supplementary Figure 11 | The high-resolution transmission electrn microscopy (HRTEM) images of the cycled spinel a) Co_3O_4, b) $Li_{0.25}Co_{2.75}O_4$, c) $Li_{0.5}Co_{2.5}O_4$, d) $Li_{0.75}Co_{2.25}O_4$, and e) $LiCo_2O_4$.

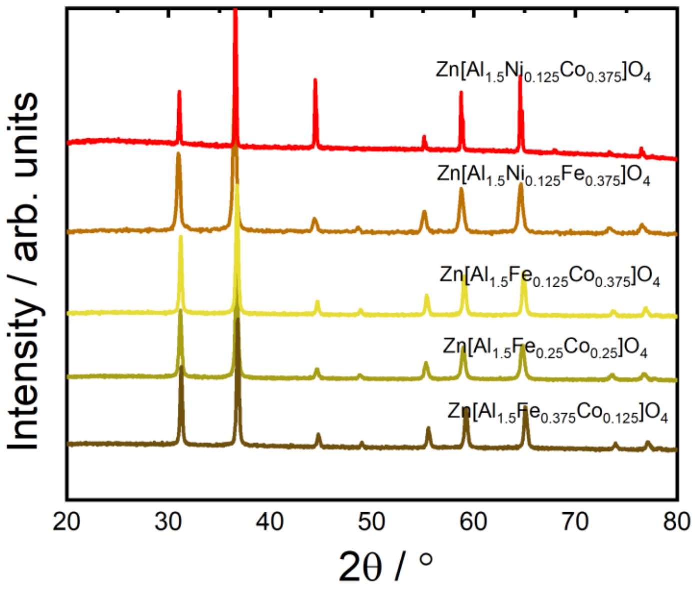

Supplementary Figure 12 | The X-ray diffraction (XRD) patterns of the prepared spinel pre-catalyst $Zn[Al_{1.5}Ni_{0.125}Co_{0.375}]O_4$, $Zn[Al_{1.5}Ni_{0.125}Fe_{0.375}]O_4$, $Zn[Al_{1.5}Fe_{0.125}Co_{0.375}]O_4$, $Zn[Al_{1.5}Fe_{0.25}Co_{0.25}]O_4$, and $Zn[Al_{1.5}Fe_{0.375}Co_{0.125}]O_4$.

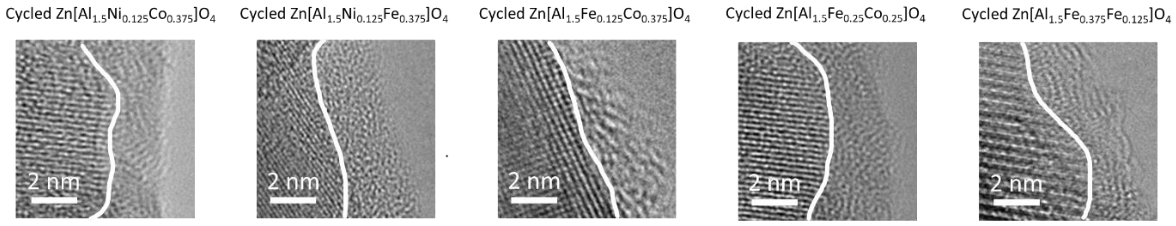

Supplementary Figure 13 | TEM images of the fully cycled spinel Zn[$Al_{1.5}Ni_{0.125}Co_{0.375}$]$O_4$, Zn[$Al_{1.5}Ni_{0.125}Fe_{0.375}$]$O_4$, Zn[$Al_{1.5}Fe_{0.125}Co_{0.375}$]$O_4$, Zn[$Al_{1.5}Fe_{0.25}Co_{0.25}$]$O_4$, and Zn[$Al_{1.5}Fe_{0.375}Co_{0.125}$]$O_4$.

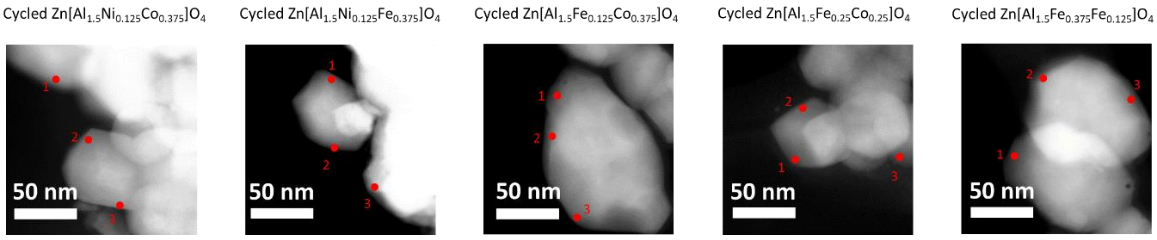

	Co	Ni
1	75.3	24.7
2	76.7	23.3
3	74.7	25.3

	Fe	Ni
1	74.4	25.6
2	76.6	23.4
3	75.6	24.4

	Fe	Co
1	25.8	74.2
2	23.3	76.7
3	23.4	76.6

	Fe	Co
1	49.2	50.8
2	49.6	50.4
3	50.3	49.7

	Fe	Co
1	74.1	25.9
2	74.6	25.4
3	76.3	23.7

Supplementary Figure 14 | The point scan results of the fully cycled spinel Zn[$Al_{1.5}Ni_{0.125}Co_{0.375}$]$O_4$, Zn[$Al_{1.5}Ni_{0.125}Fe_{0.375}$]$O_4$, Zn[$Al_{1.5}Fe_{0.125}Co_{0.375}$]$O_4$, Zn[$Al_{1.5}Fe_{0.25}Co_{0.25}$]$O_4$, and Zn[$Al_{1.5}Fe_{0.375}Co_{0.125}$]$O_4$.
